# Self-management of multiple long-term conditions: A systematic review of the barriers and facilitators amongst people experiencing socioeconomic deprivation

**DOI:** 10.1371/journal.pone.0282036

**Published:** 2023-02-21

**Authors:** Abi Woodward, Nathan Davies, Kate Walters, Danielle Nimmons, Fiona Stevenson, Joanne Protheroe, Carolyn A. Chew-Graham, Megan Armstrong

**Affiliations:** 1 Research Department of Primary Care and Population Health, University College London, London, United Kingdom; 2 School of Medicine, Keele University, Staffordshire, United Kingdom; Patan Academy of Health Sciences, NEPAL

## Abstract

**Background:**

Multiple long-term conditions are rising across all groups but people experiencing socioeconomic deprivation are found to have a higher prevalence. Self-management strategies are a vital part of healthcare for people with long-term conditions and effective strategies are associated with improved health outcomes in a variety of health conditions. The management of multiple long-term conditions are, however, less effective in people experiencing socioeconomic deprivation, leaving them more at risk of health inequalities. The purpose of this review is to identify and synthesise qualitative evidence on the barriers and facilitators of self-management on long-term conditions in those experiencing socioeconomic deprivation.

**Methods:**

MEDLINE, EMBASE, AMED, PsycINFO and CINAHL Plus were searched for qualitative studies concerning self-management of multiple long-term conditions among socioeconomically disadvantaged populations. Data were coded and thematically synthesised using NVivo.

**Findings:**

From the search results, 79 relevant qualitative studies were identified after the full text screening and 11 studies were included in the final thematic synthesis. Three overarching analytical themes were identified alongside a set of sub-themes: (1) Challenges of having multiple long-term conditions; prioritisation of conditions, impact of multiple long-term conditions on mental health and wellbeing, polypharmacy, (2) Socioeconomic barriers to self-management; financial, health literacy, compounding impact of multiple long-term conditions and socioeconomic deprivation, (3) Facilitators of self-management in people experiencing socioeconomic deprivation; maintaining independence, ‘meaningful’ activities, support networks.

**Discussion:**

Self-management of multiple long-term conditions is challenging for people experiencing socioeconomic deprivation due to barriers around financial constraints and health literacy, which can lead to poor mental health and wellbeing. To support targeted interventions, greater awareness is needed among health professionals of the barriers/challenges of self-management among these populations.

## Introduction

Long-term health conditions are defined as health problems that require ongoing management over at least a year that cannot currently be cured [1]. Globally, approximately one in three adults have multiple long-term conditions (MLTCs) [2], meaning they have two or more long-term conditions. The prevalence of MLTCs is predicted to rise in England, from 54% in 2015 to 68% in 2035 [3]. There is a need to identify ways to improve health and social care support for people with MLTCs. Understanding the experiences of people living with MLTCs is said to be critical, especially as populations age and the demand for services increases [4].

Socioeconomic deprivation, often referred to as socioeconomic status, includes a range of interacting characteristics that contribute to inequalities and disadvantage. A systematic review found people experiencing socioeconomic deprivation had a 60% higher prevalence of having a long-term health condition and a 30% increase in severity of the condition compared with those that are less socioeconomically deprived [5]. Furthermore, whilst MLTCs are rising across all groups, people living in the most deprived areas are almost twice as likely to have two or more long-term conditions, which occurs on average 10–15 years earlier compared to those living in the most affluent areas[5]. The reasons for this are unclear, with major socioeconomic inequalities existing even when accounting for common risk factors (e.g., smoking, diet, exercise, alcohol consumption) [6]. In England, the unmet need for help among older adults in the most deprived areas has been reported to be at least double that of those in the least deprived areas [1]. Similarly, in the United States (US), people living in socioeconomically deprived areas experience greater health inequalities [7]. For minorities such as Hispanics and others that are living on low incomes, the US health insurance system poses further barriers to accessing healthcare and the availability of resources [8].

Supporting people to self-manage their MLTCs is shown to be associated with improved health outcomes in a variety of health conditions [9]. Self-management refers to an individual’s ability to manage the symptoms, treatment and psychological impacts and lifestyle changes inherent in living with MLTCs [10]. Self-management therefore involves taking a proactive approach to managing health conditions such as accessing preventative services [11]. However, the management of MLTCs is challenging due to the heterogenous nature of conditions leading to an increase in primary care visits, hospital admissions and social care costs [12]. In addition, most self-management interventions are designed for single long-term conditions [13] and are less effective in people experiencing socioeconomic deprivation, to the extent it has been suggested they run the risk of exacerbating health inequalities [14]. In a report published in 2021, people with MLTCs highlighted the need for research on more holistic and integrated care approaches that prioritises psychosocial issues including the promotion of independence [15].

This systematic review builds upon existing evidence by presenting a thematic synthesis of experiences and perceptions from people with MLTCs who are socioeconomically disadvantaged. The aim of this systematic review is to explore how self-management of MLTCs can be optimised in people experiencing socioeconomic deprivation. The objective is to identify and synthesise evidence on the barriers and facilitators of self-management on long-term conditions in those experiencing socioeconomic deprivation.

## Methods

This systematic review uses a thematic synthesis methodology which was chosen based on the descriptive nature of qualitative studies [16]. By only including qualitative studies, connections can be made between relevant studies to provide an enhanced understanding of the barriers and facilitators of self-management on long-term conditions in those from experiencing socioeconomic deprivation. The review is informed by ENTREQ guidelines [17] and reported according to the PRISMA-equity guidelines [18] and guidance for thematic synthesis [16]. The review protocol is registered on the PROSPERO database (08/11/2021 CRD42021289674). Available from: https://www.crd.york.ac.uk/prospero/display_record.php?ID=CRD42021289674.

### Inclusion and exclusion criteria

Studies were included if they:

- Used qualitative methods in their approach to data collection and analysis
- Included adults over 18 years of age experiencing socioeconomic deprivation (with a proxy or quantifiable measure) with two or more long-term conditions (MLTCs)
- Explored the self-management of MLTCs

Studies were excluded if:

- Data could not be separated to identify the perspectives of those experiencing socioeconomic deprivation
- We were unable to obtain full text in English
- The papers were review articles, editorials, or conference proceedings
- Studies did not focus on self-management of MLTCs

### Search strategy

Database searches were conducted in MEDLINE, EMBASE, AMED, PsycINFO and CINAHL Plus. Databases were searched for key terms and Medical Subject Headings (MeSH) of self-management and variations of the terms ‘long-term conditions’ and ‘socioeconomic deprivation (e.g., socioeconomic status/position) were included, without date or language restrictions. Please see S1 Table for example search strategy. All article titles and abstracts were screened by two reviewers (AW and MA) independently and excluded if they did not meet the inclusion criteria. Full text papers were then screened by the first reviewer (AW) and all were checked by a second reviewer (MA). The eligibility of papers was discussed by the wider review team (CCG, ND, DN, JP, FS, KW).

### Quality assessment

A quality assessment of the literature was carried out using the Critical Appraisal Skills Programme (CASP) tool [19]. This checklist consists of ten questions which look at the results of the studies, their validity (i.e., suitability of methodological approaches used to obtain them), and how valuable and/or transferral the results are. Studies were reviewed based on the results of the quality assessment, but no studies were excluded based on quality. Utilising the CASP method highlighted the range in quality of the studies and whether recruitment and data analysis techniques were appropriate. These factors have been taken into consideration during the presentation of results.

### Thematic synthesis

The articles were analysed thematically to identify the main themes across all studies. Thematic synthesis involves three stages: the coding of text line-by-line; the development of descriptive themes; and the generation of analytical themes [16]. The results sections of each article were imported into NVivo 12 software by the first author (AW) who coded each line of text to develop a high-level coding framework around the self-management (as defined previously) of MLTCs and the barriers and facilitators to self-management among people experiencing socioeconomic deprivation. The coding framework was discussed by the wider review team (MA, CCG, ND, DN, JP, FS, KW) and then refined further as AW identified similarities and differences across the 11 studies to create descriptive themes. The descriptive themes were organised into ‘parent’ and ‘child’ nodes within NVivo to generate analytical themes based on the research question.

## Results

**Fig 1:**
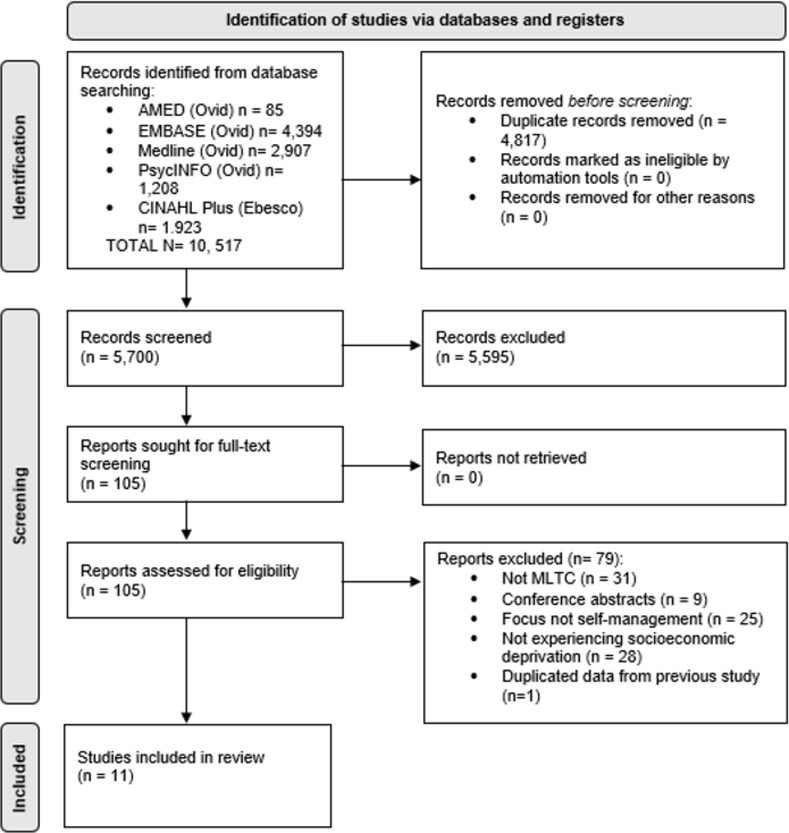
PRISMA flow diagram.

From the systematic search, 79 relevant qualitative studies were identified after the full text screening. Of these, 11 eligible articles focused on MLTCs [20–30]. A flow diagram of the review process is provided in Fig 1.

### Study characteristics

All participants from across the 11 studies had characteristics associated with socioeconomic deprivation including low income, unemployment, low educational attainment, living in an area of high deprivation. One of the studies [21] also included data from participants living in areas low social deprivation which falls out of scope for this particular review. None of the studies utilised specific measurements of socioeconomic deprivation. The studies were conducted in Australia (n = 1), Canada (n = 1), South Africa (n = 2), the United Kingdom (n = 2), and the United States (n = 5). Many of the studies reviewed did not offer a clear definition of self-management.

All of the studies used one-to-one interviews as the data collection method with one study [20] conducting two interviews with each participant. Five of the studies [20, 21, 24, 28, 29] conducted interviews in participants’ own homes, five were conducted in a room at a community, healthcare/clinic setting [23, 25–27, 30] and one study did not specify a location [22]. The participant characteristics from the studies reviewed are presented in Table 1.

**Table 1:** Primary study characteristics (n-11).

Author, year, country	Title	Aim	Study location	Detail of multi-morbidity	Study population	Socioeconomic deprivation characteristics of participant sample	Self-management definition	Analysis method	Key findings
Bardach et al., 2011, United States	The role of Social Support in Multiple Morbidity: Self- Management among rural residents	To explore how vulnerable rural residents described social support in the context of self-management for multiple chronic conditions.	Rural Appalachia, Kentucky, US	2 to 6 chronic conditions (excluding colorectal cancer). Most common combination: arthritis and high blood pressure (49%), high blood pressure and high cholesterol (41%), high blood pressure and heart disease (36%). 4.7 health conditions reported on average.	41 people (29 women, 12 men) aged 50 to 76 years old with two to six chronic conditions that required fairly extensive self-management. Ethnicity: All were White.	Authors stated all participants had low income but did not provide details. Unemployment was high (83%) and limited literacy levels reported but no details provided. All were patients at medical practices in communities with low socioeconomic and health-related indicators. Authors stated the area had high rates of poverty, isolation and poor health.	Not stated	Content/thematic analysis	Participants revealed an overall reluctance to lean on family support unless in extreme circumstances (or for more auxiliary needs). Greater reliance on providers to support medical needs/provide medical knowledge. Medicalisation of needs offered as explanation for lower reliance on family support i.e., preference to manage on own in first instance due to disadvantage within the community.
Coventry et al., 2014, UK	Capacity, responsibility, and motivation: a critical qualitative evaluation of patient and practitioner views about barriers to self-management in people with multimorbidity	To explore patient and practitioner views on factors influencing engagement in self-management in the context of multimorbidity.	Areas of high and low social deprivation in Greater Manchester, UK.	Two or more of: coronary heart disease, diabetes mellitus, osteoarthritis, chronic obstructive pulmonary disease and depression.	20 people with two or more (of five exemplar) long term conditions, aged 52 to 88 years old. Ethnicity: unknown. 20 practitioners from general practices.	Authors defined socioeconomic deprivation by the Index of Multiple Deprivation (IMD—calculated by ranking the 32,844 small areas in England i.e., from 0 to 32,844. The lower the score, the higher the deprivation). Participants lived in areas that had an IMD score of between 5.39 to 66.68	The care taken by individuals towards their own health and well-being.	Thematic analysis	Socioeconomic deprivation negatively impacted on three main factors relating to self-management: 1) capacity, 2) responsibility, 3) motivation. There was a correlation between living in area of high deprivation and holding doctor as responsible for managing and monitoring their health. Interpretive capacity of patients to self-manage MLTC is potentially an important precursor to responsibility and motivation (might be a critical target for intervention).
El-Mallakh, 2007, United States	Doing My Best: Poverty and Self-Care Among Individuals With Schizophrenia and Diabetes Mellitus	Impact of poverty on self-care of diabetes	US (exact location unknown)	Diagnosis of either schizophrenia (including paranoid, disorganized, and residual subtypes) or schizoaffective disorder and that of Type 1 or Type 2 diabetes.	11 people (5 women, 6 men) with comorbid schizophrenia and diabetes, aged 18 to 72 years old. Ethnicity: European American and African American.	Authors stated all were living in ’poverty’ but no clarification/data provided. 64% were unemployed.	Not stated	Constant comparison method	Mental illness resulted in unemployment, reliance on disability income, poverty, and material deprivation. Limited financial resources and material deprivation interfered with access to the resources necessary for adequate diabetic self-care. Treatment from mental health providers needs to consider the living situations of individuals with schizophrenia.
Hardman et al., 2021, Australia	Multimorbidity and its effect on perceived burden, capacity and the ability to self manage in a low-income rural primary care population: A qualitative study	To explore, in a low-income population with common comorbidities, how the specific demands of multimorbidity affect burden and capacity as defined by the Cumulative Complexity Model.	Rural community in Victoria, Australia	At least two chronic physical health conditions e.g., diabetes, back pain, arthritis, heart or lung conditions	13 people (6 women and 7 men) aged 47 to 72 years with 3–10 health conditions each. Ethnicity: unknown.	Authors stated that participants were clients of health centres offering services to low income and socially disadvantaged populations (no clarification provided). Self-report scales show most incomes were ’equivalent to Australian poverty line’ (A$22,000p/a).	Not stated	Framework analysis Combined analysis Grounded theory analysis	Conditions associated with functional impairment had the greatest influence on capacity. Physical, psychological and financial capacities were inseparable, but rarely addressed or understood holistically. Financial insecurity had an ’overwhelmingly’ negative effect on burden and capacity. Few people had treatment plans for their conditions. Loss of employment affected motivation for, and adherence to, self-management of conditions.
Leach and Schoenberg, 2008, United States	Striving for Control: Cognitive, Self-Care, and Faith Strategies Employed by Vulnerable Black and White Older Adults with Multiple Chronic Conditions	To increase understanding of how older adults attempt to manage multi-morbidity and retain control of their health.	Urban or suburban Kentucky, US.	At least two chronic, co-occurring illnesses or multi-morbidity. Most common reported: high blood pressure, arthritis, diabetes.	41 people (35 women, 6 men) aged 55 to 84 years with at least 2 chronic conditions. Ethnicity: white n = 11, black n = 26, other n = 4.	Authors stated that approx. one-third of participants had low income (< US$10,000/£7,600) Half of the remaining sample earned less than US$30,000/£23,000. 85% were ’not employed’ and 90% had no higher education (US college degree).	The activities individuals, families, and communities undertake with the intention of enhancing health, preventing disease, and restoring health.	Content analysis	Strong desire for individuals to remain in control of their health by employing a wide range of strategies including cognitive structuring techniques, self-care activities, and faith orientations. Self-care activities included taking medication(s), dieting, exercising, modifying activities, and regularly visiting the doctor. With the exception of faith orientations, there were no race/ethnicity differences in the self-care strategies used.
Matima et al., 2018, South Africa	A qualitative study on the experiences and perspectives of public sector patients in Cape Town in managing the workload of demands of HIV and type 2 diabetes multimorbidity	To explore the barriers to managing chronic conditions.	Informal township of Khayelitsha Cape Town, South Africa (community health centre).	HIV and Type 2 diabetes (T2D)	10 people (5 women, 5 men) with HIV and T2D aged 35 to 65 years. Ethnicity: black Xhosa speaking South Africans. 6 healthcare workers	Authors stated that 70% of participants lived in informal housing, 40% accessed a communal water supply, and 74% were on low income (approx. R2300/£160 per month).	Not stated	Thematic content analysis	Some participants were unable to meet their daily needs for self-management due to financial constraints. Demands on nutritional requirements, pill burden and stigma varied due to capacity factors e.g., health literacy, economic resources, social support. Improvements needed: integration of chronic services, consolidated guidelines for healthcare workers, educational materials for patients, improved information systems and income for patients.
Merdsoy et al., 2020, Canada	Perceptions, needs and preferences of chronic disease self-management support among men experiencing homelessness in Montreal	To explore the perceptions, needs and preferences for chronic disease self- management and self-management support among men experiencing homelessness.	Homeless shelter in Montreal, Quebec.	Any type of chronic disease or physical or mental co-morbidities. Most had multi-morbidity. Most common conditions reported: chronic pain, chronic respiratory diseases, diabetes	18 people (homeless). All men. Aged 32 to 65 years with a chronic disease. Ethnicity: Majority Canadian (plus 1 from Ethiopia and 1 from El Salvador).	Authors stated that all participants were experiencing homelessness. 17 were housed in the emergency shelter and 1 in the community. 78% had low annual income (less than CA$20,000/£12,000).	Relates to the tasks that an individual must undertake to live well with one or more chronic conditions	Inductive thematic analysis	Self-management perceived as important. Emotional self-management was most challenging. Three vulnerable groups were identified i.e., those with no social networks, severe physical symptoms, and/or co-morbid mental illness. Preferred mode of delivery for self-management support was through consistent contacts with healthcare providers and peer-support initiatives. Complex challenges relating to homelessness e.g., emotional self-management, multiple vulnerabilities and barriers to forming relationships with healthcare professionals must be addressed.
Murphy, et al., 2015, South Africa	A qualitative study of the experiences of care and motivation for effective self-management among diabetic and hypertensive patients attending public sector primary health care services in South Africa	To explore patients’ current experiences of chronic care, as well as their motivation and capacity for self-management and lifestyle change.	Residential areas in Cape Town South Africa	Diabetes and hypertension (both n = 7, additional morbidities e.g., HIV, gout, breast cancer and arthritis n = 12).	22 people (16 women, 6 men) aged 30 to 75 years. Ethnicity: A mix of Xhosa and Afrikaans speaking South Africans.	Authors state participants were experiencing socioeconomic deprivation and selected from health centres in areas of deprivation but no measurement provided. There was low educational attainment among most participants (high school education n = 11, primary school education only n = 6,)	Not stated	Content analysis	Effective self-management was impeded by poor health literacy, lack of self-efficacy and perceived social support; majority reported not having received adequate information, counselling or autonomy support from their healthcare providers. Current approach to chronic care largely fails to meet patients’ motivation needs leading to anxiety and frustration. Training, resources and tools are needed to enable healthcare providers in South Africa to adopt a more patient-centred approach.
O’Brien et al., 2014, UK	The ‘everyday work’ of living with multimorbidity in socioeconomically deprived areas of Scotland	To explore the ways in which people living in areas of high socioeconomic deprivation manage multimorbidity.	Deprived areas of Scotland, UK.	Any multi-morbidity. Most common conditions reported: depression (n = 9), diabetes (n = 8), hypertension (n = 4).	14 people (8 women and 6 men) aged 44 to 64 years. Ethnicity: unknown.	Authors stated that participants lived in areas of high socioeconomic deprivation (among top 15% most deprived).	Not stated	Thematic analysis Grounded theory analysis	Everyday life work is an important self-management component for those in deprived areas and is commonly impaired, especially in those with mental health problems. Deprivation, especially when combined with mental health problems impacted on the ability to manage multiple problems, having access to few social and material resources (and how these were perceived). Most struggled with the amount of work required to establish a sense of normalcy in everyday life.
Schoenberg et al., 2011, United States	Appalachian Residents’ Experiences With and Management of Multiple Morbidity	To improve understanding of how vulnerable rural residents experience and manage multi-morbidity.	Rural Appalachia, Kentucky, US (central Appalachian County)	Two or more chronic illnesses: heart disease or hypertension (90%), arthritis (80%), type 2 diabetes (75%), cancer (10%), stroke (10%), other illnesses (65%).	20 people (17 women, 3 men), aged 41 years or over (mean age was 55, exact age range unknown). Ethnicity: White (95%)	Authors stated that participants were experiencing socioeconomic deprivation. Clarification provided in relation to study location i.e., socioeconomic deprivation and high poverty rates. Participants self-reported as having ’just enough money to get by’ or ’not enough money to make ends meet’.	Some explanation e.g., an individual’s attempt to prevent, contain, or manage illnesses on their own or in conjunction with advice received from health care professionals, family members, or other personal relations	Iterative thematic analysis	Under resourced communities experience conditions and treatment burdens that exceed their personal and resource capacities, resulting in constrained choices. Numerous challenges were identified around self-management in a rural, under resourced context e.g., limitations attached to financial resources and general accessibility of resources reported. Strategic methods of self-management included prioritising certain conditions and management strategies and drawing heavily on assistance from informal and formal sources.
Schoenberg et al., 2009, United States	“It’s a toss up between my hearing, my heart, and my hip”: Prioritizing and Accommodating Multiple Morbidities by Vulnerable Older Adults	Explore how people manage multiple conditions	Urban or suburban areas in the south-eastern United States	Multi-morbidity: hypertension (93%), arthritis (78%), and diabetes (44%). Other common chronic conditions included cancer (27%), stroke (10%).	41 people (35 women, 6 men) aged 55 to 99 years. Ethnicity: 26% African American, 11% White, Other 4%.	Authors stated the study was focused on participants experiencing socioeconomic deprivation, but no clarification provided. 50% earned below US$30,000/£23,000. Participants self-reported as having ’just enough money to get by’ or ’not enough money to make ends meet’.	As above	Iterative thematic analysis	Heart disease, diabetes, and the combined effects of having MLTCs were the most cause for concern among individuals. Participants spent the most time and money on arthritis and diabetes. Due to financial demands, participants used resources strategically to meet their self-management needs. Enhanced formal care coordination, increased use of technological innovations, and understanding elders’ priorities are necessary to improve self-care/management and quality of life.

### Quality assessment

The CASP tool highlighted that some studies lacked detail about the methods used [22, 28] and several failed to explain the relationship between researchers and participants [20–22, 28–30]. The latter is of great importance within qualitative research to address issues of subjectivity and power imbalance [31]. The quality assessment also highlighted some issues due to vague or missing information, resulting in a ‘can’t tell’ score. For example, in relation to data collection procedures, one paper [22] references that the information is contained in an earlier publication but did not elaborate. There were also two papers that did not provide sufficient details relating to ethical considerations, beyond stating the study had been approved [20, 24]. See S2 Table for full quality assessment of the studies.

### Data synthesis results

The data presented in this section were generated from a set of analytical themes as highlighted in Table 2.

**Table 2:** Relationship between analytical themes and sub-themes.

Analytical themes	Sub-themes
Challenges of having multiple long-term conditions	Prioritisation of conditions
	Impact of multiple long-term conditions on mental health and wellbeing
	Polypharmacy
Socioeconomic barriers to self-management	Financial
	Health literacy
	Compounding impact of multiple long-term conditions and socioeconomic deprivation
Facilitators of self-management in people experiencing socioeconomic deprivation	Maintaining independence
	‘Meaningful’ activities
	Support networks

#### Challenges of multiple long-term conditions

In 10 of the 11 studies reviewed, the challenges associated with having, and managing MLTCs were discussed. The socioeconomic challenges experienced by participants typically stemmed from a lack of financial security including access to limited monetary resources. The challenges encountered were comparable across all studies but due to their international nature, participants living in countries such as the United States (US) and Australia had additional structural challenges relating to health systems and more specifically, the cost of medication, medical equipment, and some support provision which had to be paid for.

### Prioritisation of conditions

Two studies [23, 29] describe how participants prioritised the management of certain conditions over others. In an Australian-based study [23], two participants were said to rate depression as their most important condition while another prioritised obesity because of its impact on their mental health. Symptoms of depression and mental health problems were recognised as a catalyst for worsening other health conditions and impacted broadly on self-management practices:

*…it’d be so much easier if I just had one health problem I could work on and not have multiple problems… I got really bad a few months ago… I just stopped taking everything… I went into a really deep depression and couldn’t be bothered doing a thing*. (p.9) [23]

Another study which was based in the US [29], reported that that juggling several conditions was more time consuming for people than management of a single condition. Participants typically prioritised conditions based on those that were most worrisome such as conditions that threatened functional decline. Due to having MLTCs, people struggled to find the time for self-care, but they also experienced difficulties affording medication and medical equipment; especially those not covered by Medicare (a federal health insurance programme for people aged 65 or older). One participant with six long-term conditions discussed the financial implications:

*I need a new hearing aid*, *but we’ve just gotten him [husband] two new ones*, *so you can’t do it all*. *Medicare does not pay for glasses for older people or hearing aids…I don’t think that’s right*, *but they don’t [pay for them]*. (p.9) [29]

### Impact of multiple long-term conditions on mental health and wellbeing

The mental and psychological consequences of MLTCs among people experiencing socioeconomic deprivation featured in seven studies. One participant reported being unable to work because of her ill-health and described how her deterioration and inability to engage in everyday activities impacted on her mental wellbeing:

*Sometimes I sit and cry*. *I do*. *I sit and cry ‘cause I think ‘God’s sake you’re only 50 years of age*. *How did it come to this that you’re in so much pain*? *That you’ve got all these things you’ve got to do*? *And you’ve got all these illnesses*. *Just one after the other’*, *you know*, *‘everything seems to be happening’*. *I can’t make sense of it*. (p.6) [28]

The co-existence between physical and mental health, and the impact upon self-management was discussed in seven studies [21, 23, 25–28, 30]:

*…as you get older like I want to be fitter in myself and then… these little conditions*, *they stop you doing things*, *and then your motivation*, *if you’re feeling down and you’re depressed*, *then your motivation’s not there*. (p.7) [21]

*I get depressed because things don’t seem to happen quickly enough for me and I get upset that I can’t do things so I don’t eat*, *I stop taking my meds*, *I self-harm*… *things like that* (p.8) [23]

### Polypharmacy

In relation to the numerous medications needed to manage MLTCs, polypharmacy was reported as a concern in two of the studies [23, 29]. The specific challenges associated for people experiencing socioeconomic deprivation related to the costs incurred from having to take multiple medications:

*When I’m charged through [Medicaid managed care plan]*, *they charge one dollar for each prescription*, *and I take like 20–25 medications*… *that’s $30 per month*, *and I cannot afford that*, *and because of that*, *I’m having to pick and choose which medication to take and which medication to leave because I can’t afford to buy them*, *and it’s causing a lot of health problems*… (p.54) [22]

While the above example was not an issue for all participants, those included in the US and Australian based studies described the issues surrounding medical costs. For one participant who experienced several changes to his medication because of the side-effects experienced, the financial outlay of multiple visits to his doctor was a challenge:

*I paid $71 for a bottle of medicine*. *She [doctor] said I’m sorry but I’ve got to change this medicine*. *Said it’s too much for you*. *If the fluid gets too much for you then it makes you heart jump real fast*. *You have to get up in the night and everything cause it’s pounded too bad*. *Then I go back to the doctor and she said stop taking that*. *I want you to take this*. (p.8) [29]

#### Socioeconomic barriers to self-management

***Financial***. Financial barriers associated with low-income, insecure income, loss of employment/occupation and the cost of self-management were some of the common themes across the studies reviewed. Seven out of the 11 studies [21–23, 25, 27, 29, 30] described the financial difficulties and constraints experienced by people with MLTCs. One UK-based study suggested that for people who rely financially on state benefits, a lot of time and energy is spent ‘making ends meet’ rather than seeking out opportunities to enhance self-management practices:

*They’re only giving me £14 a week to live on*. *Out of that £14 I’ve got to pay £17 a week for water and heating*. *That’s another thing that does your head in because how are you supposed to live…*? *It’s playing on your mind all the time*. (p.5) [21]

Socioeconomic deprivation was present in various guises but most typically through lack of income. The studies referenced above revealed the difficulties that many participants faced when trying to manage their finances alongside the needs of their MLTCs. Struggling with transport costs was one such issue highlighted by a US-based study:

*I want to go to the clinic when I get some money*, *but I’m broke*…*I can get there*, *but I don’t know how I’m going to get back*. (p.54) [22]

Other participants described the challenges associated with having multiple medical appointments to attend which included the time management involved [29], a lack of access to local health facilities [28] and the costs incurred for attending appointments [22, 23]:

*…the psychologist that I’m seeing I…pay out-of-pocket to see her…I have to think about what don’t I get done that week do I not pay my phone or power…* (p.8) [23]

Another US-based study described how a participant with hypertension, arthritis, hyperlipidaemia, and diabetes had attributed two heart attacks to his inability to afford medication. He had since been able to access a federally qualified health clinic:

*Without this place [the clinic]*, *I wouldn’t be able to have Plavix [medication]*, *and the last two heart attacks I’ve had*, *they’ve both been when I couldn’t afford the medication…After about two months off my [medication] and the others*, *I ended up having a heart attack*. (p.604) [30]

While medication is just one way to manage health conditions, an ability to afford medicine is shown to have detrimental consequences in some cases, subsequently creating further barriers to self-management. Throughout five of the studies, the impact that financial constraints had upon diet was also highlighted, along with the ability to afford food which in turn, impacted poorly upon managing health conditions. A US-based study highlighted how conflicting financial demands led to difficult decisions over being able to afford to eat or not:

*To tell you the truth*, *a lot of times*, *I don’t eat breakfast*…*You gotta stretch it*, *you know*…*I’ll get back to eating breakfast when things get better*, *but right now I gotta pay my electric bill*. (p.54) [22]

Many participants not only struggled to afford food but found it increasingly difficult to accommodate the type of diet needed to manage specific conditions. As such, some participants were unable to follow the dietary guidance provided by healthcare professionals and nutritionists. One participant explained more about the cost implications of a specialist diet and how their different conditions required different dietary approaches:

*The coeliac disease—it’s tough and expensive with my other health issues*. *I have to be really careful about what I eat*. *I can’t eat wheat and really anything that might be processed could be a problem*. *So I got to do special things*, *like going to the health food store to buy all of these different grains*. *But that really costs me a bundle and it makes it hard to manage my diabetes*, *which requires a different diet…* (p8) [29]

*Health literacy*. In relation to understanding health information, five of the studies highlighted participants’ ability to engage with information about their health conditions, how to increase their understanding, and how to obtain more information where it was not readily available. Examples of the latter included asking questions when visiting a healthcare professional [20, 25], researching health conditions on the internet and/or through books [27] and taking notes during a medical appointment to remember what was said [24]:

*I write everything down…I take a notepad with me and*, *and whatever he [the GP] says*, *I write down at that point… maybe I’m not hearing exactly what he’s saying so when he gives me instructions*, *I write it down and then I read it back to him*. (p.391) [24]

Authors of one of the UK-based studies [21] suggest however, that people from deprived backgrounds who lack financial resources may be less able to invest in learning about, and enhance, self-management practices. Four studies revealed multiple examples of low health literacy among participants, with three of these also indicating poor health communication from health care professionals:

*When I came to the clinic*, *tests were done and the doctor just recorded a lot of things in the folder*. *They didn’t really explain anything to me*, *so even today; I really don’t know why I have this thing or what to do*. *I am worried*. (p.4) [27]

The cognitive work required to understand how treatments and conditions interacted could be burdensome [23]. Some participants reported receiving a lack of information during medical appointments which impacted their ability to understand their condition, often resulting in a lack of agency:

*I really don’t understand*. *The clinic guys wouldn’t be able to tell me about HIV*. *I was told its just dirty blood*, *that my blood is dirty… I was given a huge book to read*… *I was just told there is no cure; that this is something I have to accept now*. *There’s only medication that can just keep it*. (p.11) [25]

Low health literacy was therefore underpinned by cognitive challenges which added complexity to an individuals’ capacity to self-manage MLTCs. One participant believed that diabetes could be cured after several months of treatment:

*I believe the doctor was lying to me when she said it’s just a normal thing*, *because a normal thing reaches a point when it gets out of the body*. *But I find that its three months now and I am carrying on and on… So far*, *I am taking my medication*, *but after 4 months (if I am not cured) I will drink Aloe Vera if necessary*. (p.4) [27]

#### Compounding impact of multiple long-term conditions and socioeconomic deprivation

One study highlighted the negative impact that living in an area of deprivation can have upon health. For example, poor life expectancy can become normalised for some people from deprived areas because ‘they are surrounded by other people that are ill and neighbours that are ill’ [21] (p.7). Consequently, living in an area of high deprivation could have a psychological impact:

*[My area] is a place where they send you to die really*. *They should put a fence round it*. *They send you there to forget about you*. (p.6) [28]

Feelings such as those demonstrated above, could exacerbate the mental health conditions that participants had alongside other long-term conditions. Living with MLTCs meant for some, that they were faced with the reality of no longer being able to work due to a decline in functional capacity. Loss of occupation could lead to complex mental and psychological issues, which in turn led to some participants experiencing social isolation [21, 26, 28]. As such, social isolation was seen to further reduce participants’ capacity to engage in self-management practices [21] since it is entangled with emotional and mental health problems. In addition, the financial implications of being unable to work could counteract people’s ability to self-manage:

*I’m on disability*, *I can’t work*, *I can’t get no job*…*I got no money to buy the diabetic pills*, *the last time I took them was a month ago*. *I don’t have $4 a month to pay for them*. (p.54) [22]

For one participant living in Australia, an inability to work due to a functional impairment which led to a new disability, also led to them having to sell their house:

*I’ve lost my house that was the main thing…I nearly had it paid off [but] I had no insurance because I had shoulder operations before and they wouldn’t give me income insurance*… (p.8) [23]

#### Facilitators of self-management in people experiencing socioeconomic deprivation

Nine out of the 11 studies [21–28, 30] described aspects of participants’ lives that acted as facilitators of self-management among people with MLTCs. These included independence, employment, informal support from family and peer networks, physical activity, and diet.

### Maintaining independence

All studies discussed the capacity that individuals had for self-managing their conditions. Eight of the studies referred to the personal responsibility or self-reliance that participants felt towards maintaining their own health. For example, some were cautious of relinquishing tasks or chores to other people before necessary, to maintain some level of control over daily life:

*Well I don’t get a lot of help from anybody cause I live by myself and I take care of my apartment and I do my groceries and things and some things the kids will do but not too much*. *‘Cause the way I feel about it*, *the longer either physically and mentally*, *the longer I push to do these things*, *the longer I will be able to do them as I age in life*. *That’s the reason I like doing things for myself*. (p.9) [29]

Similarly, some participants resisted asking for help in order to maintain some control or a sense of personal identity and pride:

*I like to be strong and I’ve also took care of myself*. *I’ve never asked naebody [nobody] for help*. *Ever*. *I help everybody else*. *But I never ask for help… Now I just don’t want to ask for help*. *I just go without*. *I would just stay in*. (p.7) [28]

Self-reliance could also be an impetus for gaining more knowledge about self-management:

*We got to understand that we got to manage our own health because we care more about our health than others… As a patient you are responsible for your own self*. (p.6) [20]

### *‘*Meaningful’ activities

Working part-time was a favourable option among two participants of an Australian-based study [23], despite it being physically demanding and exacerbating their chronic pain. The study found that while employment provided greater financial security to participants compared to those in receipt of unemployment benefits from the state, employment was also beneficial to people’s mental wellbeing:

*…with depression people handle it in different ways I keep busy I work I do things if I can’t work what happens I go downhill*…*as soon as I stop doing things I go downhill*. (p.9) [23]

Correspondingly, as alluded to earlier, giving up employment or being of an age where one no longer worked, could result in experiences of social isolation and other mental health issues [28] that were counterproductive against self-management. It was important therefore in the absence of work to engage in other meaningful activities such as gardening or housework since these were linked to positive self-management practices:

*I’ve got to be doing something*. *I’ve got to be tidying up*. *I’ve got to make sure my house is the way I want*. *No I wouldn’t sit in my own filth*, *no*. *I’ve got to do my housework whether it kills me or not*. (p.6) [28]

### Support networks

The theme of social (or informal) support appeared in nine of the studies [20–23, 25–27, 29, 30] but was demonstrated to varying degrees as some participants preferred to only lean on family or other support networks when necessary. The most typical examples focused upon physical activity such as exercise [21, 23–25, 27, 29, 30] and engaging with support groups and/or peer support, including faith groups [21, 25–29].

Peer support as a form of self-management intervention was shown to be vital for people experiencing homelessness, helping them to manage the emotional challenges of both MLTCs and homelessness:

*…it’s good to have people to talk to…even if you knew people were just around… it just made you feel*, *sort of*, *better*. (p.1428) [26]

Some people were able to implement and sustain lifestyle changes through other types of activity which in turn, helped them engage with a group of people with important commonalities:

*I am part of an exercise group*. *I started last year and now I go every Thursday*. *I am really happy about joining*. *We are about 15 and everyone has a chronic illness*, *so we share ideas*. (p.6) [27]

As demonstrated above, being around people who were in a relatable situation or just having someone to talk to, could provide mental health benefits [25]. While physical activity could be a way to connect to others with MLTCs some found exercise difficult or challenging to undertake; especially when experiencing chronic pain [23, 29]. This review shows however, that access to support provision varies across localities and could be offered as a free referral from a healthcare professional or incur a cost. Referrals to provision such as an exercise programme was a barrier to self-management for one participant who was struggling financially:

*My doctor tells me it would be good for my sugar [diabetes] to take some exercise*, *but it’s hard—the rent*, *food*, *my medicine*, *it adds up*. *Joining a club’s not going to work*. (p.9) [29]

Self-management was also facilitated at times by access to distributed health literacy since some participants had access to family members that helped provide emotional reassurances as well as typically providing tangible assistance:

*She [my daughter] knows a lot and what to do in medical situations*. *So she was always there every day and every evening*. *And I have another daughter who is an accountant*, *so she did a good job of keeping up with all of my paying the bills because my husband doesn’t know a lot about writing a check or keeping up with the bills*. *So I do have great support from family*. (p.9) [20]

Some participants concerned about their financial circumstances and how they could support their self-management, were able to ask family members for financial support. Examples of financial support included getting money from family to top-up low-income/benefits [23] and family members paying for prescriptions and buying specialist food items [29] such as getting help with buying healthy food as described below:

*Mom and Dad help me*, *and that’s definite*, *they’ll help me; if I can’t buy the food*, *they’ll buy it for me*. *They’ve already said*, *We’d help you if you don’t make it*, *you know*, *but try and make it*, *because we’re not going to be around all the time*…*and they’re 78 and 77 years old right now…* (p.55) [22]

## Discussion

This systematic review has highlighted the specific challenges that people experiencing socioeconomic deprivation face when self-managing MLTCs. Mental health conditions along with the prioritisation of conditions and polypharmacy all pose challenges for people experiencing MLTCs. Mental and physical health are typically exacerbated by worries and anxieties relating to money which can be worsened by financial factors such as the cost of, or inability to afford multiple medications. The decline or loss of functional capacity brought about by the presence of MLTCs also has negative impact on people’s mental health serving to impact other areas of life. Some people therefore prioritise the management of mental health symptoms such as those relating to depression since these can act as a barrier to self-management practices, as found in previous research [32], serving to worsen other long-term conditions over time. Consequently, having MLTCs to manage was worrisome for people experiencing socioeconomic deprivation, with the lack of personal control (perceived or actual) that they may have over their own lives being an important factor [33]. Moreover, those with pre-existing health restrictions are at higher risk of losing their jobs and will likely experience deterioration in their mental and physical health due to unemployment [34–36]. The subsequent loss of routine, structure and sense of purpose [37], further compound feelings of hopelessness and social isolation [38],

The studies reviewed, in part, reflect some of the differences across global health systems which were linked to financial barriers. The US for example, provide healthcare through a combination of private health insurance and public health coverage (Medicare, Medicaid) while in the UK and Canada, there is a universal healthcare programme. In Australia, it is primarily funded through the public Medicare programme but in South Africa, private and public health systems exist in parallel. The results from the US studies in particular [20, 22, 29, 30] highlight how some people with MLTCs that also experience socioeconomic deprivation, may have difficulty accessing additional provision such as an exercise programme as a form of self-management, due to the costs incurred. The compounding impact of MLTCs and socioeconomic deprivation meant that some also struggled to afford medication and medical equipment, and at times experienced the financial implications of visiting the doctor. Despite the international nature of the studies, there was at times, commonality among participants surrounding the barriers to self-management. Financial insecurity and access to limited financial resources impacts greatly upon quality of life and have associated mental health and wellbeing implications for people living with MLTC, especially among those experiencing loss of occupation/employment due to deteriorating health.

Some people also lack the knowledge and/or understanding of healthy behaviours to benefit their condition e.g., a healthy diet. Health literacy therefore appears to play a significant role in facilitating healthy behaviour, yet this review suggests people with lower health literacy also experience higher levels of anxiety about their health. Health literacy has been defined as ‘a person’s knowledge, motivation and competences to access, understand, appraise, and apply health information in order to make judgments and take decisions in everyday life concerning healthcare, disease prevention and health promotion to maintain or improve quality of life during the life course’ [39]. The concept of distributed health literacy on the other hand ‘refers to the way in which health literacy is distributed throughout a group of individuals or a community’ [40] such as turning to family and is more likely to be used where low health literacy exists. People with low or poor health literacy are nonetheless, evidenced as being less able to successfully manage chronic disorder, as well as incurring higher healthcare costs [41]. People experiencing socioeconomic deprivation who also have low health literacy are still poorly understood, despite advancements in this area [42]. Consequently, the review indicates that lower health literacy has the potential to reinforce existing health inequalities [43] and experiences of self-reliance and personal responsibility will vary accordingly.

Engaging in meaningful activities such as paid work are shown to facilitate self-management practices due to the mental health benefits and financial security that stem from employment. This review suggests therefore that loss of employment is a catalyst to health inequalities and wider physical and mental health issues [36]. The review also suggests that people experiencing socioeconomic deprivation may at times draw upon informal support such as family to provide more tangible assistance which helps with self-management. However, there is strong evidence to suggest that people with MLTCs who also experience socioeconomic deprivation prefer to be self-reliant and therefore rely more upon health providers to support their medical rather than auxiliary needs, holding family and other informal support networks (if present) in reserve. As the review shows, this can be problematic for individuals with lower levels of health literacy. These results intersect with themes surrounding personal control but also relate to a sense of identity and pride. As such, it is typical for people experiencing socioeconomic disadvantage to acquire ways of coping that protect their social identity, and in the UK, there is a burgeoning body of literature on the inter-related themes of poverty, shame, stigma, and feelings of unreservedness [44, 45]. Maintaining some self-reliance in relation to the management of health is one way for people with MLTCs to take some control, especially when faced with socioeconomic disadvantage. It is necessary however, to consider how self-reliance correlates with health literacy.

## Strengths and limitations of the review

This review is the first systematic review to our knowledge that evidences the compounding issues of self-managing MLTCs when experiencing socioeconomic deprivation. The primary papers identified have allowed us to present an international perspective on this topic. However, most studies identified a socioeconomically disadvantaged sample by focusing on areas of deprivation or asking participants to ‘self-report’; therefore, the extent of deprivation has not been objectively measured (e.g., be collecting data on income). The participant samples also included people from both urban and rural settings which need to be taken into consideration when reviewing the results due to the availability of healthcare services. As noted, only one study that was included in the review [21] focused on individuals living in areas of both high and low deprivation. An exploration of experiences among those with low levels of socioeconomic deprivation have not therefore been captured. Nevertheless, the review does provide important insights and evidence surrounding the self-management of MLTCs among people experiencing socioeconomic deprivation.

## Implications for clinical practice and future research

More research is needed to better understand what improvements can be made for people with MLTCs, especially since most interventions are designed for single conditions [13]. The review highlights the need for greater understanding of how individuals with MLTCs prioritise their needs and how liable these may be to change over time; both due to condition severity and socioeconomic deprivation. There is strong evidence to suggest that people with MLTCs that experience socioeconomic deprivation rely more upon health providers to support their medical needs, to maintain some independence. As such, greater awareness is needed among health professionals of the barriers/challenges of self-management among these populations. Future research could explore the experiences of populations with low levels of socioeconomic deprivation as well as focusing on strategies to promote health literacy among socioeconomically disadvantaged groups; the issues of which are inextricably tied to poor literacy and numeracy skills [41]. Interventions that are targeted to support the mental health issues among people with MLTCs are also needed, with a view to preventing the worsening of other long-term conditions.

## Conclusion

People experiencing socioeconomic deprivation who live with MLTCs prioritise certain conditions over others, and report difficulty managing multiple medication, which can lead to poor mental health and wellbeing. In addition, the self-management of MLTCs is challenging for socioeconomically disadvantaged populations due to barriers including financial constraints and health literacy. However, maintaining independence, engaging in meaningful activities and having a strong support network helped overcome these barriers. More recognition of the impact that socioeconomic deprivation has on people’s ability to self-management from healthcare professionals and researchers will lead to better self-management support and interventions.

## Supporting information

S1 Table This is the example database search. (DOCX)

S2 Table This is the full CASP quality appraisal. (DOCX)

S1 Checklist (PDF)
