# Supplementary material for: Self-management of multiple long-term conditions: A systematic review of the barriers and facilitators amongst people experiencing socioeconomic deprivation
Source: PLoS One. 2023 Feb 21;18(2):e0282036. doi: 10.1371/journal.pone.0282036 (PMC9942951; doi:10.1371/journal.pone.0282036)
Supplement: S1 Table — (DOCX) [file pone.0282036.s001.docx]

| **#** | **CONCEPT** | **Sub-group** | **Query** | **AMED** | **EMBASE** | **MEDLINE** | **PsycINFO** |  |
| --- | --- | --- | --- | --- | --- | --- | --- | --- |
| 1 | **Self-management** | N/A | "self-manag*".ab,ti. | 863 | 32,961 | 23,066 | 10,931 |  |
| 2 |  |  | "self-monitor*".ab,ti. | 153 | 12,370 | 8,885 | 6,382 |  |
| 3 |  |  | "self-care".ab,ti. | 1,195 | 28,859 | 20,494 | 10,519 |  |
| 4 |  |  | "self-help".ab,ti. | 369 | 8,752 | 6,926 | 8,609 |  |
| 5 |  |  | "self-maintenance".ab,ti. | 15 | 835 | 700 | 378 |  |
| 6 |  |  | self-maintain.ab,ti. | 0 | 68 | 38 | 10 |  |
| 7 |  |  | 1 or 2 or 3 or 4 or 5 or 6 | 2,500 | 79,116 | 56,689 | 35,274 |  |
| 8 | **Long-term conditions** | Multi-morbidity | "multiple chronic conditions".ab,ti. | 24 | 1,631 | 1,331 | 359 |  |
| 9 |  |  | co-morbid*.ab,ti. | 316 | 68,933 | 29,769 | 7,164 |  |
| 10 |  |  | co?morbid*.ab,ti. | 1,878 | 322,602 | 187,462 | 55,401 |  |
| 11 |  |  | multi?morbid*.ab,ti. | 61 | 8,069 | 6,198 | 1,089 |  |
| 12 |  |  | multi-morbid*.ab,ti. | 7 | 1,567 | 955 | 194 |  |
| 13 |  |  | polychronic*.ab,ti. | 0 | 22 | 24 | 165 |  |
| 14 |  |  | poly?morbid*.ab,ti. | 2 | 625 | 397 | 16 |  |
| 15 |  |  | poly-morbid*.ab,ti. | 0 | 22 | 18 | 3 |  |
| 16 |  |  | "co-occurring condition*".ab,ti. | 5 | 553 | 403 | 364 |  |
| 17 |  |  | "concurrent condition*".ab,ti. | 2 | 413 | 290 | 83 |  |
| 18 |  |  | 8 or 9 or 10 or 11 or 12 or 13 or 14 or 15 or 16 or 17 | 2,259 | 392,271 | 222,354 | 63,478 |  |
| 19 |  | Chronic illness | "chronic illness*".ab,ti. | 730 | 23,118 | 16,927 | 9,779 |  |
| 20 |  |  | "chronic disease*".ab,ti. | 1,231 | 100,197 | 71,903 | 11,865 |  |
| 21 |  |  | "chronic condition*".ab,ti. | 550 | 28,757 | 21,120 | 5,973 |  |
| 22 |  |  | NCD*.ab,ti. | 20 | 12,780 | 8,027 | 662 |  |
| 23 |  |  | "noncommunicable disease*".ab,ti. | 28 | 3,942 | 3,429 | 340 |  |
| 24 |  |  | "non-communicable disease*".ab,ti. | 39 | 11,004 | 8,785 | 790 |  |
| 25 |  |  | "noncommunicable illness*".ab,ti. | 0 | 9 | 7 | 1 |  |
| 26 |  |  | "non-communicable illness*".ab,ti. | 0 | 12 | 11 | 1 |  |
| 27 |  |  | "long-term disease*".ab,ti. | 22 | 5,762 | 3,588 | 144 |  |
| 28 |  |  | "long-term illness*".ab,ti. | 32 | 879 | 779 | 359 |  |
| 29 |  |  | "long-term condition*".ab,ti. | 102 | 2,816 | 2,113 | 700 |  |
| 30 |  |  | 19 or 20 or 21 or 22 or 23 or 24 or 25 or 26 or 27 or 28 or 29 | 2,614 | 176,214 | 126,382 | 28,182 |  |
| 31 |  | Specific conditions-NCDs | diabetes.ab,ti. | 4,365 | 872,646 | 573,632 | 31,243 |  |
| 32 |  |  | "cardiovascular disease*".ab,ti. | 1,256 | 275,588 | 191,031 | 11,920 |  |
| 33 |  |  | CVD.ab,ti. | 181 | 65,321 | 41,605 | 3,115 |  |
| 34 |  |  | "coronary artery disease".ab,ti. | 349 | 142,176 | 89,762 | 2,028 |  |
| 35 |  |  | "chronic respiratory disease".ab,ti. | 77 | 3,295 | 2,325 | 104 |  |
| 36 |  |  | "chronic obstructive pulmonary disease".ab,ti. | 1,292 | 77,229 | 53,293 | 2,347 |  |
| 37 |  |  | COPD.ab,ti. | 1,404 | 96,680 | 50,971 | 1,714 |  |
| 38 |  |  | asthma.ab,ti. | 1,759 | 226,965 | 155,603 | 7,615 |  |
| 39 |  |  | cancer.ab,ti. | 14,348 | 2,655,179 | 1,885,284 | 64,642 |  |
| 40 |  |  | cancers.ab,ti. | 794 | 424,662 | 294,492 | 4,648 |  |
| 41 |  |  | dementia.ab,ti. | 2,602 | 172,890 | 119,556 | 67,345 |  |
| 42 |  |  | epilepsy.ab,ti. | 663 | 159,653 | 110,905 | 33,557 |  |
| 43 |  |  | stroke.ab,ti. | 9,383 | 428,109 | 267,852 | 34,528 |  |
| 44 |  |  | multiple sclerosis.ab,ti. | 2,108 | 126,551 | 81,318 | 16,182 |  |
| 45 |  |  | Parkinson's.ab,ti. | 1,720 | 142,023 | 97,471 | 27,944 |  |
| 46 |  |  | "motor neuron disease".ab,ti. | 39 | 6,712 | 4,603 | 949 |  |
| 47 |  |  | neurodegenerative.ab,ti. | 401 | 131,565 | 98,026 | 20,987 |  |
| 48 |  |  | arthritis.ab,ti. | 3,840 | 274,309 | 185,921 | 5,460 |  |
| 49 |  |  | "inflammatory arthropathies".ab,ti. | 4 | 613 | 376 | 2 |  |
| 50 |  |  | osteoporosis.ab,ti. | 1,170 | 111,471 | 71,586 | 1,920 |  |
| 51 |  |  | "liver disease".ab,ti. | 300 | 146,688 | 92,148 | 1,627 |  |
| 52 |  |  | cirrhosis.ab,ti. | 150 | 147,772 | 96,545 | 1,160 |  |
| 53 |  |  | 31 or 32 or 33 or 34 or 35 or 36 or 37 or 38 or 39 or 40 or 41 or 42 or 43 or 44 or 45 or 46 or 47 or 48 or 49 or 50 or 51 or 52 | 43,710 | 5,665,918 | 3,924,749 | 294,133 |  |
| 54 |  | Specific conditions-Long term infectious | HIV.ab,ti. | 1,867 | 423,586 | 330,382 | 54,651 |  |
| 55 |  |  | "human immunodeficiency virus".ab,ti. | 329 | 98,785 | 90,387 | 5,999 |  |
| 56 |  |  | AIDS.ab,ti. | 2,235 | 180,893 | 154,951 | 36,405 |  |
| 57 |  |  | "acquired immunodeficiency syndrome".ab,ti. | 93 | 17,008 | 16,302 | 749 |  |
| 58 |  |  | 54 or 55 or 56 or 57 | 3,481 | 542,095 | 430,993 | 71,928 |  |
| 59 |  | Specific conditions- mental health | depression.ab,ti. | 6,891 | 505,662 | 368,524 | 260,011 |  |
| 60 |  |  | anxiety.ab,ti. | 4,787 | 310,956 | 220,001 | 206,821 |  |
| 61 |  |  | "anxiety disorder".ab,ti. | 213 | 24,236 | 17,678 | 17,482 |  |
| 62 |  |  | schizophrenia.ab,ti. | 1,013 | 154,859 | 114,261 | 105,369 |  |
| 63 |  |  | "schizophrenic disorder*".ab,ti. | 10 | 1,505 | 1,083 | 1,309 |  |
| 64 |  |  | bipolar.ab,ti. | 345 | 97,489 | 67,414 | 41,308 |  |
| 65 |  |  | "bipolar disorder".ab,ti. | 134 | 44,120 | 28,745 | 26,201 |  |
| 66 |  |  | "bipolar affective disorder".ab,ti. | 16 | 3,066 | 2,136 | 2,001 |  |
| 67 |  |  | "panic disorder".ab,ti. | 89 | 12,100 | 9,382 | 10,484 |  |
| 68 |  |  | 59 or 60 or 61 or 62 or 63 or 64 or 65 or 66 or 67 | 10,571 | 868,326 | 637,895 | 503,207 |  |
| 69 |  | ***Subtotal*** | 18 or 30 or 53 or 58 or 68 | 57,655 | 7,119,971 | 5,035,541 | 855,488 |  |
| 70 | **SES** | Social determinant concept | "social inequalities".ab,ti. | 7 | 2,842 | 2,700 | 1,437 |  |
| 71 |  |  | "health inequalities".ab,ti. | 26 | 5,659 | 5,189 | 1,732 |  |
| 72 |  |  | "health inequit*".ab,ti. | 15 | 3,045 | 2,867 | 877 |  |
| 73 |  |  | "social gradient".ab,ti. | 3 | 847 | 745 | 265 |  |
| 74 |  |  | "social determinants".ab,ti. | 33 | 10,402 | 8,811 | 3,330 |  |
| 75 |  |  | 70 or 71 or 72 or 73 or 74 | 82 | 21,177 | 18,741 | 7,098 |  |
| 76 |  | SES terms | socio-economic.ab,ti. | 269 | 45,735 | 34,970 | 14,404 |  |
| 77 |  |  | socioeconomic.ab,ti. | 658 | 124,187 | 101,323 | 48,869 |  |
| 78 |  |  | social-economic.ab,ti. | 151 | 6,068 | 4,970 | 3,591 |  |
| 79 |  |  | SES.ab,ti. | 126 | 25,729 | 18,329 | 18,241 |  |
| 80 |  |  | "social class".ab,ti. | 79 | 9,471 | 8,772 | 10,098 |  |
| 81 |  |  | "working class".ab,ti. | 15 | 1,355 | 1,284 | 4,233 |  |
| 82 |  |  | "social status".ab,ti. | 63 | 7,691 | 6,384 | 7,638 |  |
| 83 |  |  | "social position".ab,ti. | 7 | 1,213 | 1,090 | 1,438 |  |
| 84 |  |  | "low-status".ab,ti. | 12 | 930 | 884 | 1,821 |  |
| 85 |  |  | "social* depriv*".ab,ti. | 21 | 3,097 | 2,317 | 1,051 |  |
| 86 |  |  | "material* depriv*".ab,ti. | 5 | 746 | 628 | 298 |  |
| 87 |  |  | "socially excluded".ab,ti. | 10 | 302 | 240 | 413 |  |
| 88 |  |  | "social* exclusion".ab,ti. | 37 | 2,445 | 2,021 | 3,325 |  |
| 89 |  |  | 76 or 77 or 78 or 79 or 80 or 81 or 82 or 83 or 84 or 85 or 86 or 87 or 88 | 1,365 | 204,491 | 164,135 | 99,333 |  |
| 90 |  | Vulnerable group terms (not SES specific) | vulnerable*.ab,ti. | 823 | 125,116 | 97,953 | 40,247 |  |
| 91 |  |  | disadvantaged.ab,ti. | 175 | 17,241 | 15,189 | 14,964 |  |
| 92 |  |  | underserved.ab,ti. | 180 | 15,911 | 11,549 | 5,525 |  |
| 93 |  |  | marginali#ed.ab,ti. | 129 | 7,341 | 6,500 | 10,232 |  |
| 94 |  |  | marginali#ation.ab,ti. | 56 | 2,902 | 2,615 | 4,843 |  |
| 95 |  |  | "less affluent".ab,ti. | 4 | 515 | 427 | 215 |  |
| 96 |  |  | under-represent*.ab,ti. | 8,210 | 6,522 | 4,686 | 1,910 |  |
| 97 |  |  | "hard-to-reach".ab,ti. | 11 | 2,893 | 2,337 | 1,076 |  |
| 98 |  |  | 90 or 91 or 92 or 93 or 94 or 95 or 96 or 97 | 9,455 | 173,435 | 137,125 | 75,703 |  |
| 99 |  | Subgroup: education | "low* educat*".ab,ti. | 117 | 22,120 | 17,267 | 7,059 |  |
| 100 |  |  | "less educat*".ab,ti. | 80 | 7,905 | 6,518 | 3,550 |  |
| 101 |  |  | "education* level".ab,ti. | 411 | 48,565 | 34,985 | 16,657 |  |
| 102 |  |  | "education* status".ab,ti. | 52 | 6,712 | 4,907 | 1,602 |  |
| 103 |  |  | "education* attainment".ab,ti. | 83 | 11,426 | 9,778 | 7,607 |  |
| 104 |  |  | "higher educat*".ab,ti. | 418 | 23,989 | 19,128 | 31,447 |  |
| 105 |  |  | 99 or 100 or 101 or 102 or 103 or 104 | 1,038 | 98,308 | 75,145 | 61,041 |  |
| 106 |  | Subgroup -occupation (inc. unemployment) | unemploy*.ab,ti. | 536 | 25,994 | 20,267 | 15,830 |  |
| 107 |  |  | occupation.ab,ti. | 1,716 | 41,194 | 33,853 | 14,866 |  |
| 108 |  |  | "insecure employment".ab,ti. | 1 | 35 | 36 | 33 |  |
| 109 |  |  | 106 or 107 or 108 | 2,232 | 66,181 | 53,406 | 30,424 |  |
| 110 |  | Subgroup- housing (inc. homelessness | "housing".ab,ti. | 607 | 37,666 | 32,393 | 18,394 |  |
| 111 |  |  | "permanent supportive housing".ab,ti. | 5 | 150 | 151 | 128 |  |
| 112 |  |  | "social housing".ab,ti. | 3 | 628 | 497 | 373 |  |
| 113 |  |  | "council-hous*".ab,ti. | 2 | 54 | 46 | 23 |  |
| 114 |  |  | homeless*.ab,ti. | 339 | 14,753 | 12,067 | 11,685 |  |
| 115 |  |  | 110 or 111 or 112 or 113 or 114 | 856 | 49,562 | 42,129 | 27,447 |  |
| 116 |  | Proxy: area of deprivation | deprivation.ab,ti. | 315 | 93,254 | 72,056 | 24,187 |  |
| 117 |  |  | "disadvantaged area".ab,ti. | 3 | 160 | 137 | 94 |  |
| 118 |  |  | "low-income neighbo?rhood".ab,ti. | 1 | 180 | 156 | 134 |  |
| 119 |  |  | 116 or 117 or 118 | 319 | 93,579 | 72,339 | 24,408 |  |
| 120 |  | Proxy: Income | low-income.ab,ti. | 236 | 46,511 | 39,945 | 22,626 |  |
| 121 |  |  | low-resource.ab,ti. | 33 | 9,986 | 7,706 | 1,084 |  |
| 122 |  |  | limited-resource.ab,ti. | 5 | 2,335 | 1,614 | 468 |  |
| 123 |  |  | impoverished.ab,ti. | 38 | 4,318 | 3,865 | 3,672 |  |
| 124 |  |  | indigent.ab,ti. | 14 | 2,496 | 2,203 | 504 |  |
| 125 |  |  | uninsured.ab,ti. | 29 | 12,501 | 8,794 | 1,837 |  |
| 126 |  |  | underinsured.ab,ti. | 8 | 1,141 | 774 | 174 |  |
| 127 |  |  | under-insured.ab,ti. | 90 | 121 | 56 | 19 |  |
| 128 |  |  | "social assistance".ab,ti. | 18 | 1,029 | 835 | 493 |  |
| 129 |  |  | "welfare-assist*".ab,ti. | 1 | 101 | 97 | 77 |  |
| 130 |  |  | "state-support*".ab,ti. | 13 | 700 | 599 | 488 |  |
| 131 |  |  | "state-benefits".ab,ti. | 3 | 87 | 75 | 60 |  |
| 132 |  |  | "income support".ab,ti. | 8 | 354 | 311 | 245 |  |
| 133 |  |  | subsidi#ed.ab,ti. | 27 | 3,944 | 3,101 | 1,125 |  |
| 134 |  |  | "financially disadvantaged".ab,ti. | 1 | 109 | 82 | 54 |  |
| 135 |  |  | 120 or 121 or 122 or 123 or 124 or 125 or 126 or 127 or 128 or 129 or 130 or 131 or 132 or 133 or 134 | 498 | 81,323 | 66,693 | 31,826 |  |
| 136 |  | ***Subtotal*** | 75 or 89 or 98 or 105 or 109 or 115 or 119 or 135 | 14,925 | 689,722 | 550,817 | 315,210 |  |
| 137 | **TOTAL** | | 7 and 69 and 136 | 85 | 4,394 | 2,907 | 1,208 |  |
