# Supplementary material for: Self-management of multiple long-term conditions: A systematic review of the barriers and facilitators amongst people experiencing socioeconomic deprivation
Source: PLoS One. 2023 Feb 21;18(2):e0282036. doi: 10.1371/journal.pone.0282036 (PMC9942951; doi:10.1371/journal.pone.0282036)
Supplement: S2 Table — (DOCX) [file pone.0282036.s002.docx]

**S2 Table**

| **Source paper** | **Title of paper** | **CASP checklist: Quality Assessment** | | | | | | | | | |
| --- | --- | --- | --- | --- | --- | --- | --- | --- | --- | --- | --- |
|  |  | Was there a clear statement of the aims of the research? | Is a qualitative methodology appropriate? | Was the research design appropriate to address the aims of the research? | Was the recruitment strategy appropriate to the aims of the research? | Was the data collected in a way that addressed the research issue? | Has the relationship between researcher and participants been adequately considered? | Have ethical issues been taken into consideration? | Was the data analysis sufficiently rigorous? | Is there a clear statement of findings? | How valuable is the research? |
| Bardach et al., 2011, United States (n=41) | The role of Social Support in Multiple Morbidity: Self-Management among rural residents | Yes | Yes | Yes | Yes | Yes | Can't tell | Can't tell | Yes | Yes | Research is valuable in terms of exploring reasons for support preferences and how socioeconomic disadvantage affects choices and decision making around self-management. |
| Coventry et al., 2014, UK (n=20) | Capacity, responsibility, and motivation: a critical qualitative evaluation of patient and practitioner views about barriers to self-management in people with multimorbidity | Yes | Yes | Yes | Yes | Yes | Can't tell | Yes | Yes | Yes | Research contributes to understanding around socioeconomic deprivation and self-management in relation to area, but individual levels of deprivation cannot be determined. |
| El-Mallakh, 2007, United States (n=11) | Doing My Best: Poverty and Self-Care Among Individuals With Schizophrenia and Diabetes Mellitus | Can't tell | Yes | Can't tell | Can't tell | Can't tell | No | Yes | Yes | Yes | Research is somewhat of value for identifying the financial/income barriers attached to self-care, but more detail is needed on the research design and analysis method to ensure greater research integrity. |
| Hardman et al., 2021, Australia (n=13) | Multimorbidity and its effect on perceived burden, capacity and the ability to self manage in a low-income rural primary care population: A qualitative study | Yes | Yes | Yes | Yes | Yes | Yes | Yes | Yes | Yes | Research contributes valuable findings on socioeconomically disadvantaged people and provides a robust overview of the implications to self-management. |
| Leach and Schoenberg, 2008, United States (n=41) | Striving for Control: Cognitive, Self-Care, and Faith Strategies Employed by Vulnerable Black and White Older Adults with Multiple Chronic Conditions | Yes | Yes | Yes | Yes | Yes | Can't tell | Can't tell | Yes | Yes | Good recording of self-care activities but analysis of how socioeconomic status affects this is missing. Research fails to go beyond the assumptions of poverty attached to the study population group (i.e., black participants). |
| Matima et al., 2018, South Africa (n=10) | A qualitative study on the experiences and perspectives of public sector patients in Cape Town in managing the workload of demands of HIV and type 2 diabetes multimorbidity | Yes | Yes | Yes | Yes | Yes | Yes | Yes | Yes | Yes | Provides clear links between the constraints of socioeconomic deprivation and self-management and insightful recommendations for policymakers and primary healthcare. |
| Merdsoy et al., 2020, Canada (n=18) | Perceptions, needs and preferences of chronic disease self-management support among men experiencing homelessness in Montreal | Yes | Yes | Yes | Yes | Yes | Yes | Yes | Yes | Yes | Research contributes in particular to understanding of, and the challenges surrounding emotional self-management. Detailed findings which include tables and figures are very useful for further illustrating results. |
| Murphy, et al., 2015, South Africa (n=22) | A qualitative study of the experiences of care and motivation for effective self-management among diabetic and hypertensive patients attending public sector primary health care services in South Africa | Yes | Yes | Yes | Yes | Yes | Yes | Yes | Yes | Yes | Provides useful insight into lack of health literacy, material resources and self-efficacy among participants that had low educational attainment. |
| O'Brien et al., 2014, UK (n=14) | The ‘everyday work’ of living with multimorbidity in socioeconomically deprived areas of Scotland | Yes | Yes | Yes | Yes | Can't tell | Can't tell | Yes | Yes | Yes | Provides clear links about the negative impact of deprivation on self-management. This is presented in the context of area deprivation only, yet participants were not explicitly asked about their experiences of living in very deprived areas which appears fundamental to the theme of 'everyday life work'. |
| Schoenberg et al., 2011, United States (n=20) | Appalachian Residents’ Experiences With and Management of Multiple Morbidity | Yes | Yes | Yes | Yes | Yes | Can't tell | Yes | Yes | Yes | Provides useful insight around prioritisation of certain conditions, associated management strategies and the links to resource-scarce communities. |
| Schoenberg et al., 2009, United States (n=41) | “It’s a toss up between my hearing, my heart, and my hip”: Prioritizing and Accommodating Multiple Morbidities by Vulnerable Older Adults | Yes | Yes | Yes | Yes | Yes | Can't tell | Yes | Yes | Yes | Provides useful summary of the most time consuming and costly conditions for participants and some insights around how they negotiated financial demands to meet their self-management needs. |
